# The International Vocabulary of Tinnitus

**DOI:** 10.3389/fnins.2022.887592

**Published:** 2022-05-03

**Authors:** David M. Baguley, Charlotte Caimino, Annick Gilles, Laure Jacquemin

**Affiliations:** ^1^Nottingham Biomedical Research Centre, National Institute for Health Research, Nottingham, United Kingdom; ^2^Hearing Sciences, Mental Health and Clinical Neurosciences, School of Medicine, University of Nottingham, Nottingham, United Kingdom; ^3^Nottingham University Hospitals NHS Trust, Nottingham, United Kingdom; ^4^Department of Otorhinolaryngology, Antwerp University Hospital, Edegem, Belgium; ^5^Department of Translational Neuroscience, Faculty of Medicine and Health Science, University of Antwerp, Antwerp, Belgium; ^6^Department of Education, Health and Social Work, University College Ghent, Ghent, Belgium

**Keywords:** tinnitus, terminology, language, vocabulary, qualitative research

## Abstract

Tinnitus is a common experience which can have a severe impact on ones quality of life. Whilst there have been reports of historical references to tinnitus, there has not been an international cross-sectional analysis of the vocabulary used for tinnitus. In this study, with 227 respondents (of which 53.3% experiencing tinnitus themselves), we report such an analysis of 252 words or phrases, from 42 languages and 48 countries. The results indicate that the majority of vocabulary used has a negative connotation (63%), though a small minority are positive (4%). Many words used for tinnitus in different languages are onomatopoeic—thus mimicking aspects of the percept experienced—or describe the sound (in total 42% of the vocabulary). The involvement of the ear is implied in some terminology, though other vocabulary expresses the impact. Participants experiencing tinnitus significantly differed on the codes for their proposed words or phrases (*p* < 0.001), with the code “internal suffering or irritation or intrusion” being more prevalent and the code “relate to ear” and “sound is phantom or not real or imagined” being less prevalent in this group. This research has implications not only for the vocabulary used for tinnitus in Patient Reported Outcome Measures but also, and importantly, for understanding the vocabulary and lived experiences of people with tinnitus by healthcare professionals.

## Introduction

It is well known that the English word *tinnitus* derives from the Latin verb *tinnire* (to ring). The experience of tinnitus is that of a conscious sound perception in the absence of an external auditory stimulus (Baguley et al., 2013), and this can be simple (a single tone or noise, often high frequency and strident) or complex (multiple percepts) and can vary markedly on a moment by moment basis. Tinnitus is common auditory experience, estimated at 12–30% of the adult population (Bhatt et al., 2016; Mccormack et al., 2016) (though prevalence estimates vary on the basis of the specific question posed (Mccormack et al., 2016). Moreover, the prevalence of troublesome tinnitus is lower, with a total of 5% in adults (Davis and Rafaie, 2000), and 3% in children (Rosing et al., 2016). There is no cure for tinnitus (Mcferran et al., 2019) and present treatment relies upon counseling and sound therapy (Cima et al., 2019).

The first published use of the word tinnitus in English is ascribed to Blanchards Physicians Dictionary (1693) where it appears as Tinnitus Aurium, placed between *tinea* (skin ulcers on the head) and *titillatio* (tickling) (Stephens, 1987). Feldmann (1997) indicates that the introduction of the word tinnitus to the scientific literature was within the works of Pliny the Elder (23/24-79AD) superceding the use of *sonitus* which had been used by Celsus (dates unknown: active c.30AD).

Whilst the word tinnitus is presently in common use, it has been noted that, in some languages, there are a variety of terms used to describe the experience (Feldmann, 1997). For example, Stephens (1987, 2000) observed that in French the terms *acouphènes*, *bourdonnements* (buzzing), *stifflements* (whistling), *tintements* (ringing), and *tintouins* (unpleasant noise) are in use. A number of these terms have qualities of onomatopoeia, in that the word resembles the sound experience it describes. Each of the words beginning with *tin* have a high frequency and strident quality. As such, the vocabulary for tinnitus can have different interpretations with regards to its characteristics, severity and distress that this phantom sound can cause.

Consideration of the terminology used by clinicians, patients, and the public about medical symptoms and conditions is an important element of the developing disciple of narrative medicine (Franz and Murphy, 2018), the aim being that medical linguistics can assist in understanding the lived experience of an individual. To date, there has been no research that has scoped the variety of terms used for tinnitus in languages worldwide, and indeed such a study would have been daunting until the widespread international use of the internet, and social media, and tools by which to collect data.

In the present study, the aim was to collect synonyms for tinnitus in as many languages as possible. Additionally, a review of the meaning of the terms used was proposed. The overall purpose was to scope out the international vocabulary that is in present use to describe tinnitus. Potentially this could shed light on the lived experience of people with tinnitus, but also indicate discrepancies between those and the understanding and vocabulary of clinicians and researchers. This may be of some value when instruments/metrics to determine the severity of tinnitus are designed.

## Materials and Methods

### Design

A cross-sectional study was used to examine the international vocabulary used for tinnitus using an anonymous online questionnaire which was developed for the purposes of the project. Ethical approval was obtained from the University of Nottingham Faculty of Medicine and Health Science Ethics Committee (FMHS 155-0121) and Committee for Medical Ethics of the University Hospital Antwerp (B3002021000031) prior to data collection.

### Participants

Participants were recruited online using an opportunity sampling method in which they were provided with an anonymous link to the questionnaire. The study link was advertised to participants (public and professional) on social media sites including Facebook, LinkedIn, and Twitter, and to colleagues within the University of Nottingham Hearing Sciences, University of Antwerp, and Antwerp University Hospital. Criteria for inclusion in the study was the ability to understand, read and write English language sufficiently to complete the questionnaire, the ability to complete the questionnaire online, and being an adult (age 18 years or over). A total of 239 participants met the criteria to take part in the study, however 12 of these participants did not complete key sections of the questionnaire and their data were removed. Therefore, the final sample consisted of 227 participants with complete data that was analyzed.

### Materials

A single online questionnaire was developed for the purpose of the study and hosted on Bristol Online Surveys Joint Information Systems Committee (JISC) platform. Participants were first asked to provide basic demographic information including age and occupation (if any), this was followed by questions relating to the languages they most commonly speak, the terms used for tinnitus in their language and meanings in English, the connotations of the word and finally if they experience tinnitus themselves. A detailed overview of the questionnaire can be seen in Supplementary Material 1.

### Procedure

Participants were initially directed to the study via an anonymous link. Upon clicking the link participants were presented with a page providing information about the nature of the study. This included the study aims to ensure that informed consent was obtained before taking part and inclusion/exclusion criteria. The information page also stated that participation in the study was entirely voluntary, there was no obligation to take part and participants may withdraw at any point during the study. The information page presented the participants with a statement regarding the use of their data and rights, data protection, and information on the study’s ethical approval. The contact details of the research team were provided for any further information about the study. Before consenting, participants were asked to carefully read the information and consent statements on the first page of the survey. Participants then completed the questionnaire and were presented with debrief information and contact details of the research team.

The bottom-up (inductive) thematic analysis methodology (Braun and Clarke, 2006) was followed in order to create codes for the words and phrases, which were collected in the survey, and these codes were grouped into themes (including “miscellaneous” to house codes that do not fit into the three main themes). This methodology implies that the codes are created by highlighting and making notes and that the themes identified are strongly linked to the data, instead of trying to fit it into pre-existing codes. Two researchers independently coded the data (DB and CC). Every difference was discussed and a final coding was agreed on.

Data were analyzed using SPSS statistical software version 27 (SPSS Inc., Chicago, IL, United States). In order to compare data of participants with and without tinnitus, chi-square-tests were conducted. More specifically, the themes of the words and phrases reported by these two groups were compared, as well as the attributed connotations.

## Results

### Demographics

Table 1 displays demographic information for the 227 participants. Participants ranged from 18 years to 75+ years old. The majority of participants (66.1%) had a health-related profession, of which 41% of the participants were related to Audiology. A total of 59% of participants’ professions also involved conducting research, the most common area of research was Health Care (54.5%). The majority of participants indicated they experienced tinnitus themselves (53.3%), 45.4% did not experience tinnitus, and 1.3% preferred not to say.

**TABLE 1:** Participant demographics.

**Age range of participants (years)**	***N*** **(%)**
18–24	55 (24.2%)
25–34	68 (30%)
35–44	37 (16.3%)
45–54	30 (13.2%)
55–64	23 (10.1%)
65–74	12 (5.3%)
75 +	2 (0.9%)
**Health-related profession**	***N*** **(%)**
Yes	150 (66.1%)
No	76 (33.5%)
Prefer not to say	1 (0.4%)
**Involved in research**	***N*** **(%)**
Yes	134 (59%)
No	84 (37%)
Prefer not to say	9 (4%)
**Area of research (in case involved in research)**	***N*** **(%)**
Arts	1 (0.7%)
Biological/Biomedical	6 (4.5%)
Business	3 (2.2%)
Communication	6 (4.5%)
Computer and information sciences	2 (1.5%)
Education	2 (1.5%)
Engineering	6 (4.5%)
Health care	73 (54.5%)
History	1 (0.7%)
Other	12 (9%)
Psychology	17 (12.7%)
Social Science	5 (3.7%)
**Experience tinnitus themselves**	***N*** **(%)**
Yes	121 (53.3%)
No	103 (45.4%)
Prefer not to say	3 (1.3%)

### Countries and Languages

**FIGURE 1:**
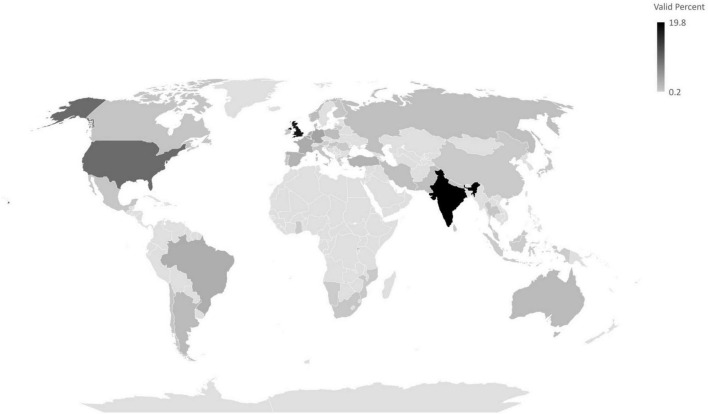
Countries identified in the survey.

Figure 1 outlines the countries in which the participants were from. A total of 48 countries were identified. A full list of the countries and their corresponding languages can be found in Supplementary Material 2. Most participants were from Europe (47.1%) and Asia (31.7%). The other continents reached were North America (11.2%), South America (5.7%), Africa (2.4%), and Oceania (1.9%).

A total 42 languages were identified, 33.5% of participants spoke English, 54.2% spoke English and one other language, 11.9% spoke English and two other languages, and 0.4% spoke English and four other languages.

### Words and Phrases Identified

**FIGURE 2:**
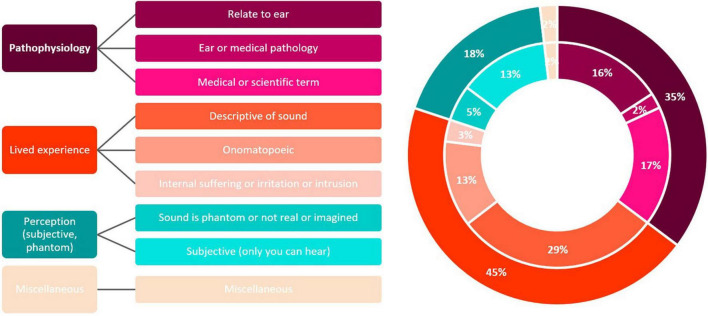
Codes and themes identified by the bottom-up thematic analysis, which was conducted by two researchers independently.

**FIGURE 3:**
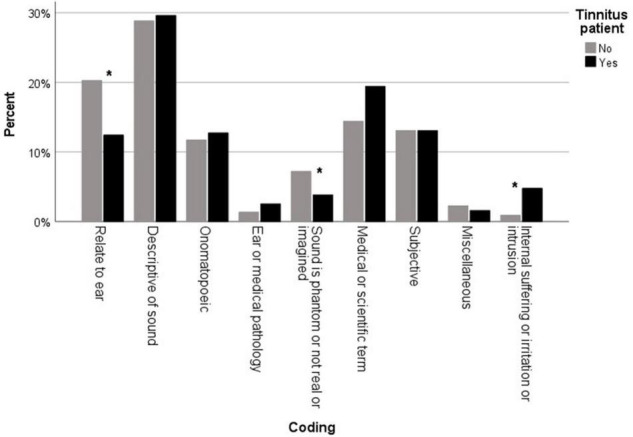
Percentage of codes identified for the vocabulary used by participants that do experience tinnitus (black) and by participants that do not experience tinnitus (gray). Significant differences (*p* < 0.05) are indicated by an asterisk (*).

A full list of words and phrases identified as synonyms for tinnitus in the different languages can be found in Supplementary Material 3. Participants were able to provide up to three words and phrases to describe tinnitus symptoms. A total of 252 words or phrases were provided. Using the bottom-up (inductive) thematic analysis methodology (Braun and Clarke, 2006), nine codes were created for the words and phrases collected in the survey and these codes were grouped into four themes (i.e., pathophysiology, lived experience, perception, miscellaneous). The codes and overarching themes are presented in Figure 2. A total of 32 differences in coding between the two researchers were discussed in order to reach this final coding. Participants experiencing tinnitus significantly differed on the codes for their proposed words or phrases (X^2^ = 52.08; *p* < 0.001; V = 0.18), with the code “internal suffering or irritation or intrusion” being more prevalent and the code “relate to ear” and “sound is phantom or not real or imagined” being less prevalent in this group (Figure 3).

**FIGURE 4:**
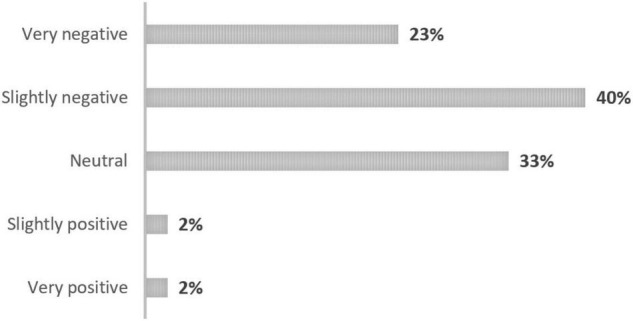
Conations of the words and phrases according to the participants themselves.

As shown in Figure 4, most of the words and phrases had a slightly negative (40%) or very negative (23%) connotation according to the participants. Only 4% of the words and phrases were slightly or very positive. There was a significant difference between participants with tinnitus or without tinnitus in terms of the connotations attributed to the words and phrases (X^2^ = 11.93; *p* = 0.018; V = 0.21), with tinnitus patients attributing more often a “very negative” connotation.

## Discussion

The overall purpose of this study was to scope out the international vocabulary that is in present use to describe tinnitus. Words or phrases for tinnitus in as many languages as possible were collected and a review of the meanings and connotations was performed. By comparing the survey results between participants that did experience tinnitus themselves and participants who did not, we wanted to shed light on the lived experience of tinnitus, but also indicate discrepancies between those and the understanding and vocabulary of clinicians and researchers.

### Lived Experience

Most meanings of the words and phrases for the tinnitus experience in different languages could be categorized as “lived experience.” Words and phrases that described the sound were most common within this theme, as for example hissing *(English)*, rauschen *(“rushing” in German)*, çınlama or çıtırtı *(“ringing” or “crackle” in Turkish)*, shabdam or kadal *(“sound/noise” or “sea” in Malayalam), dzwonienie (“ringing” in Polish)* or vez vez *(“buzzing” in Persian).* Instead of describing the sound, some words just sound similar to the referred noise (i.e., onomatopeic), as with piep *(“beep” in Dutch)* or zumbido (“buzz” in *Portuguese*). More rarely, the words or phrases were related to the internal suffering, irritation or intrusion. For example, terms as “interference” or “invasive” came up in the survey, which were often suggested by participants with tinnitus themselves, and as such, probably reflecting their own experiences. Hence, the code “internal suffering or irritation or intrusion” was more prevalent in the vocabulary of participants experiencing tinnitus.

### Pathophysiology

Within the theme of “pathophysiology,” words and phrases were often related to medical or scientific terms. More specifically, 17% of all responses were similar to tinnitus or auditory hallucinations. A note of caution is due here as the overrepresentation of healthcare professions in the sample may have led to a potential bias. Tinnitus seems also to be used in other languages, such as Dutch and Norwegian. However, some languages have their own medical term for it: acouphène (French), acúfenos *(Spanish)*, acufene or tinnito *(Italian)*, çınlama *(Turkish)*, тиннитус *(Russian)* or Miminari *(Japanese)*. Other words and phrases referred to the role of the ear, which were more prevalent in the non-tinnitus group. Most of the time the role of the ear was combined with a description of the sound (which was also classified within the “lived experience” theme): ringing in the ears *(English)*, oorsuizen *(“earsizzle” in Dutch)* and Øresus *(“earwhoosing” in Norwegian)*, ohrgeräusche *(“earsizzle” in German)*, kaan me awaz *(“sound in ears” in Hindi)*, fulzugas *(“noise in the ear” in Hungarian)*, шум B ушах *(“noise in ears” in Russian)*.

### Perception

As tinnitus is in most cases subjective and as such solely perceived by the patient, 18% of the words and phrases referred to this aspect. Similarly to tinnitus being called a “phantom sound” in English, “fantoomgeluid” is used in Dutch. However, this terminology was more often reported by non-tinnitus patients. Moreover, the subjective aspect was emphasized in responses such as “internal noise.”

### Miscellaneous

Some responses could not be categorized in one of the three main themes. For example, the response that the mother tongue did not have a word for it. One participant reported the lack of a word for it in Urdu. Another participant that spoke Sinhalese contacted us after the survey with a similar remark, and even did not know of the existence of tinnitus before the start of their PhD on tinnitus. This draws the attention toward the importance of the current research. A further study could provide the questions in the mother tongue of the participants, while the current findings may be somewhat limited by the need to understand, read and write English language sufficiently to complete the questionnaire.

This research has implications for the vocabulary used for tinnitus in Patient Reported Outcome Measures, especially as outcomes might be influenced by the terminology having a negative connotation. This study also draws attention to the understanding the lived experiences of people with tinnitus, as there were significant differences between participants experiencing tinnitus or not. Continued efforts are needed with regard to the vocabulary of tinnitus used by clinicians and researchers. Their vocabulary to be known and understood by the public and cover the wide range of tinnitus perceptions and experiences. Future research could validate the current findings by providing the survey in different languages and with respect to cultural differences.

## Data Availability Statement

The original contributions presented in the study are included in the article/Supplementary Material, further inquiries can be directed to the corresponding author/s.

## Ethics Statement

The studies involving human participants were reviewed and approved by the University of Nottingham Faculty of Medicine and Health Science Ethics Committee for Medical Ethics of the University Hospital Antwerp. The patients/participants provided their written informed consent to participate in this study.

## Author Contributions

CC organized the database. LJ performed the statistical analysis. DB and LJ wrote the first draft of the manuscript. DB, CC, and LJ wrote sections of the manuscript. All authors contributed to conception and design of the study, manuscript revision, read, and approved the submitted version.

## Author Disclaimer

Their views herein are their own and do not represent those of NIHR nor the UK Department for Health and Social Care.

## Conflict of Interest

The authors declare that the research was conducted in the absence of any commercial or financial relationships that could be construed as a potential conflict of interest.

## Publisher’s Note

All claims expressed in this article are solely those of the authors and do not necessarily represent those of their affiliated organizations, or those of the publisher, the editors and the reviewers. Any product that may be evaluated in this article, or claim that may be made by its manufacturer, is not guaranteed or endorsed by the publisher.
