# Supplementary material for: The International Vocabulary of Tinnitus
Source: Front Neurosci. 2022 May 3;16:887592. doi: 10.3389/fnins.2022.887592 (PMC9111008; doi:10.3389/fnins.2022.887592)
Supplement: Supplementary file 1 [file Table_1.DOCX]

Global vocabulary for hearing symptoms

Page 1: Study Information

Study Title: Global vocabulary for hearing symptoms

Research Team:

Professor David Baguley

Charlotte Caimino

Hearing Sciences, Mental Health and Clinical Neurosciences, School of Medicine, University of Nottingham, National Institute for Health Research, Nottingham Hearing Biomedical Research Centre, Ropewalk House, 113 The Ropewalk, Nottingham, NG1 5DU

Professor Annick Giles

Dr Laure Jacquemin

University Hospital Antwerp, Wilrijkstraat 10, B-2650 Edegem, Belgium

This study has been reviewed and given a favourable opinion by the University of Nottingham, Faculty of Medicine & Health Sciences Research Ethics Committee [FMHS REC ref no FMHS 155-01-21].

This study will aim to capture different terms used for the perception of noise in the ear or head which is not present in the external environment, and the connotations of these terms used around the globe. We aim to collate as many of these as possible and present the wide variety and disparity between countries and languages.

Thank you for your interest. You are invited to take part because you are aged over 18 years, and are able to read and write in English. Please read through this information before agreeing to participate. You can ask any questions before deciding by contacting the researchers (details below). Taking part is entirely voluntary.

What will I be asked to do?

You will be asked a series of short questions which will include providing some basic demographic information (i.e. age, occupation) and information relating the language you speak.

It should take you about 5 - 10 minutes to complete. No background knowledge is required. We would like you to answer all questions as honestly and completely as possible however if there is a question you do not want to answer then there is a ‘prefer not to say’ option. You can withdraw at any point during the questionnaire for any reason, before submitting your answers by clicking the Exit button/closing the browser. After submitting your answers it will not be possible to withdraw. You will then be presented with a Debrief page giving more information about the research topic and details of who to contact if you would like more information about the study.

What are the disadvantages of taking part?

By taking part in the study participants will give up their personal time. However, the questionnaire should not take more than 5 - 10 minutes to fill out.

What are the advantages of taking part?

Your contribution together with others will help the researchers to understand more about the different terms used in different countries around the world.

Who will know I have taken part in the study?

No one will know you have taken part in this study because we will not ask for your name or any other personal ID during this questionnaire. Your IP address will not be visible or stored by the research team because an online survey platform is being used which receives and stores an IP address but enables this detail to be filtered out before it is transferred to the research team. As with any online related activity the risk of breach is possible but this risk is being minimized by using a platform that sits on an encrypted webpage. For further information about the online survey tool security please see https://www.onlinesurveys.ac.uk/security/

What will happen to your data?

When you have clicked the submit button at the end of the questionnaire, it will be uploaded into a password protected database with a code number. The research team will not be able to see who it is from and for this reason it will not possible to withdraw the data at this point. Your data (research data) will be stored in a password-protected folder sitting on a restricted access server at the University under the terms of its data protection policy. Data is kept for a minimum of 7 years.

The results may be used in academic publications and presentations. The overall anonymised data from this study may be shared for use in future research and teaching (with research ethics approval).

The only personal data we will receive is your e-mail if you contact us to ask further questions. This will be received and handled separately from your completed questionnaire and it will not be possible to link the sets of data. Your e-mail address will only be kept as long as needed to resolve your query. It will then be destroyed. For further information about how the university processes personal data please see: https://www.nottingham.ac.uk/utilities/privacy.aspx/

Who will have access to your data?

The University of Nottingham is the data controller (legally responsible for data security) and the Chief Investigator Prof David Baguley is the data custodian (manages access to the data) and as such will determine how your data is used in the study. Your research and personal data will be used for the purposes of the research only. Research is a task that we perform in the public interest.

Responsible members of the University of Nottingham may be given access to data for monitoring and/or audit of the study to ensure it is being carried out correctly.

If you have any questions or concerns about this project, please contact the Chief Investigator:

Prof David Baguley David.Baguley@Nottingham.ac.uk

If you remain unhappy and wish to complain formally, you should then contact the FMHS Research Ethics Committee Administrator E-mail: FMHS-ResearchEthics@nottingham.ac.uk

If you are happy to proceed with taking part in the study please click next to be taken to the consent page.

If you are happy to proceed with the study please click next to be taken to the consent page.

Page 2: Consent

Study Title:

Research Team:

Professor David Baguley

Charlotte Caimino

Hearing Sciences, Mental Health and Clinical Neurosciences, School of Medicine, University of Nottingham, National Institute for Health Research, Nottingham Hearing Biomedical Research Centre, Ropewalk House, 113 The Ropewalk, Nottingham, NG1 5DU

Professor Annick Giles

Dr Laure Jacquemin

University Hospital Antwerp, Wilrijkstraat 10, B-2650 Edegem, Belgium

This study has been reviewed and given a favourable opinion by the University of Nottingham, Faculty of Medicine & Health Sciences Research Ethics Committee [FMHS REC ref no FMHS 155-0121].

Thank you for participating!

Please tick each box to continue:

Please select at least 6 answer(s).

- I confirm that I have read and understood the information on the previous page
- I confirm I can understand, read and write in English language
- I am 18 years old and/or older
- I understand that my participation is voluntary and I can end the study at any time and withdraw my data by clicking the EXIT button
- I understand that my answers are anonymous
- I understand the overall anonymized data from this study may be used in the future for research (with research ethics approval) and teaching purposes

Page 3

1) What language do you speak everyday?

<free text>

1a) In the case of multiple languages, which one did you mostly speak as a child?

<free text>

2) What country are you from?

<drop down list>

3) What is your age?

- 18-24 years old
- 25-34 years old
- 35-44 years old
- 45-54 years old
- 55-64 years old
- 65-74 years old
- 75 years or older
- Prefer not to say

4) Is your profession or study health-related?

- Yes
- No
- Prefer not to say

4a) Please specify what your profession is:

<free text>

5) Are you involved in conducting research in your profession?

- Yes
- No
- Prefer not to say

5a) Please specify from the list below your area of research:

- Health care
- Business
- Social Science
- History
- Psychology
- Biological/ Biomedical
- Engineering
- Arts
- Communication
- Education
- Computer and Information Sciences
- Other
- Prefer not to say

If you selected Other, please specify:

Page 4

Please read the below statement and answer the following questions with this statement in mind.

Hearing a sound (beep, tone, or other noise) without a corresponding external sound being present.

6) What words or phrases would you use for this experience in the language you speak everyday? If you do not know of more than one word/phrase please specify N/A for word/phrase two and three

|  | | | Select the meanings this word/phrase has: | | | | |  |
| --- | --- | --- | --- | --- | --- | --- | --- | --- |
|  |  | Can you explain briefly what this word/phrase means (in English)? | Very negative | Slightly negative | Neutral | Slightly positive | Very positive | |
| Word/Phrase 1 | <free text> | <free text> |  |  |  |  |  | |
| Word/Phrase 2 | <free text> | <free text> |  |  |  |  |  | |
| Word/Phrase 3 | <free text> | <free text> |  |  |  |  |  | |

7) Do you experience this hearing symptom yourself?

- Yes
- No
- Prefer not to say

Final page

Thank you for taking part in this study!

This study will aim to capture different terms used for tinnitus (the perception of noise in the ear without a corresponding external sound being present) and the connotations of the terms used around the globe. Often different terms are possible in one language, each with their own connotation. This results in a wide variety of phrases used, with both positive and negative meanings. We aim to collate as many of these as possible and present the wide variety and disparity between countries and languages. This vital first step may bring the field closer to a common definition of tinnitus that facilitates communication not only between different research and treatment centres around the world, but also between patients and healthcare providers.

If you have any questions or would like further information please contact the Chief Investigator Professor David Baguley: David.Baguley@nottingham.ac.uk
