# Supplementary material for: The International Vocabulary of Tinnitus
Source: Front Neurosci. 2022 May 3;16:887592. doi: 10.3389/fnins.2022.887592 (PMC9111008; doi:10.3389/fnins.2022.887592)
Supplement: Supplementary file 2 [file Table_2.DOCX]

| Argentina | Spanish |
| --- | --- |
|  | French |
| Australia | English |
|  | Vietnamese |
| Austria | German |
| Belgium | Dutch |
|  | English |
|  | Danish |
|  | Flemish |
| Brazil | English |
|  | Portuguese |
| Canada | English |
|  | Arabic |
| Chile | Spanish |
|  | English |
| China | Chinese |
| Croatia | Croatian |
|  | English |
|  | Dutch |
| Cyprus | English |
|  | Greek |
| Denmark | Danish |
|  | English |
| Finland | English |
|  | Finnish |
| France | English |
|  | French |
|  | Italian |
| Germany | Dutch |
|  | English |
|  | German |
| Ghana | English |
| Greece | Greek |
| Hungary | Hungarian |
| India | English |
|  | Bengali |
|  | Hindi |
|  | Malayalam |
|  | Oriya |
|  | Hindi |
|  | Kannada |
|  | Marathi |
|  | Tamil |
|  | Urdu |
| Indonesia | English |
|  | Indonesian |
| Iran | Farsi |
|  | Persian |
|  | Kurdish |
| Ireland {Republic} | English |
| Israel | English |
| Italy | Italian |
|  | English |
|  | Spanish |
| Japan | Japanese |
| Lebanon | French |
|  | Arabic |
|  | English |
| Lithuania | Lithuanian |
| Malaysia | English |
|  | Tamil |
|  | Malay |
| Malta | English |
|  | French |
|  | German |
| Mexico | Spanish |
| Mozambique | Gujarati |
|  | English |
| Namibia | English |
| Nepal | English |
|  | Hindi |
|  | Nepali |
| Netherlands | Dutch |
|  | English |
| Norway | Norwegian |
| Pakistan | English |
|  | Urdu |
|  | Punjabi |
| Poland | English |
|  | Polish |
| Portugal | Portuguese |
| Romania | Romanian |
|  | English |
| Russian Federation | Russian |
|  | English |
| Rwanda | English |
|  | French |
|  | Kinyarwanda |
| Singapore | English |
|  | Bahasa Indonesia |
|  | Arabic |
| South Africa | English |
| Spain | Catalán |
|  | Spanish |
|  | English |
| Sri Lanka | Sinhalese |
| Thailand | Thai |
| Turkey | Turkish |
|  | English |
| UK | English |
|  | Spanish |
| United States | English |
|  | German |
