# Supplementary material for: The International Vocabulary of Tinnitus
Source: Front Neurosci. 2022 May 3;16:887592. doi: 10.3389/fnins.2022.887592 (PMC9111008; doi:10.3389/fnins.2022.887592)
Supplement: Supplementary file 3 [file Table_3.DOCX]

| Catalán | Acúfenos |
| --- | --- |
|  | Pitidos |
|  | Tinnitus |
| Catalan/ Spanish/ English | Acúfen |
|  | brunzit |
|  | gemec |
|  | Sons acúfens |
|  | Tinnitus |
|  | xiulet |
| Chinese | 嗡嗡声（像机器轰鸣） |
|  | 嗡嗡声（像电流声） |
| Croatian/ English/ Dutch | Tinitus |
|  | zvonjava u ušima |
| Danish | Hylen |
|  | Ringen |
|  | Tinnitus |
| Dutch | een tuut horen |
|  | fantoomgeluid |
|  | fluit/piep in je oor |
|  | gefluit in de oren |
|  | geluid illusie |
|  | hallucinatie |
|  | Irritant |
|  | oorsuizen |
|  | piep |
|  | ruis horen |
|  | Tinnitus |
| Dutch/ English | Phantom noise |
|  | Piep in de/je oren |
|  | Ringing in the ears |
|  | Tinitus |
|  | Tinnitus |
| English | a noise in your/my head |
|  | A ring in my ear |
|  | Annoying |
|  | Auditory hallucination |
|  | Auditory hallucinations |
|  | background sound |
|  | Beeping |
|  | Brain making sounds |
|  | Buzzing |
|  | buzzing in ears |
|  | Clicking |
|  | Constant noise |
|  | Constant steam coming out of a boiling kettle. |
|  | Deafness |
|  | Don't know why sounds plays in my ear |
|  | ears ringing |
|  | Experiencing tinnitus. |
|  | Habituated |
|  | Head buzz |
|  | Head noise |
|  | Hear a ringing or buzzing |
|  | Hearing a constant beep even in silence |
|  | Hearing any sound that isn't from around you |
|  | Hearing disorder |
|  | Hearing Loss |
|  | Hearing things |
|  | Heart beat noise |
|  | High pitched whistling |
|  | Hiss |
|  | hissing in the ears |
|  | I hear a sound that no one hears |
|  | I hear a variation of tones |
|  | I know but would not say 'phantom sound' |
|  | In the ear sound comes in its own |
|  | Illusion |
|  | Interference |
|  | Internal noise |
|  | Invasive |
|  | Irritating |
|  | Louder repetitive high note |
|  | Low bleep or puffing noise |
|  | Medium high frequency gas oven hissing |
|  | Mistake (simply based on the description you provide) |
|  | Moolal |
|  | Musical hallucinations |
|  | My ear is singing |
|  | Noises in your head |
|  | Numbness |
|  | Pain |
|  | Reactive tinnitus |
|  | Ringing |
|  | ringing in ears |
|  | ringing in my ears |
|  | Ringing in the ear |
|  | Ringing in the ears |
|  | Ringing in your ears |
|  | Ringing sensation |
|  | Ringing tone lasting 5 seconds sounds like telephone engaged signal but continuous |
|  | ringing/buzzing/humming in ears |
|  | Roaring |
|  | Rumbling |
|  | Screeching |
|  | Symphony |
|  | The ear makes internal noise |
|  | Throbbing |
|  | Tinitus |
|  | tinnitis |
|  | Tinnitus |
|  | Tone |
|  | Tormenting despairing |
| English, Hindi, Nepali | Kaan karaune |
|  | Tinnitus |
| English/ Bahasa Indonesia/ Arabic | Tinitus |
|  | Tinnitus |
|  | طنين الأذن |
| English/ Bengali | Bhalo |
|  | Kothai |
|  | Ma |
| English/ Danish/ Flemish | Hearing a noise that’s not there |
|  | Tinnitus |
| English/ Finnish | Hallusinaatio |
|  | Kuulohäiriö |
|  | Tinnitus |
| English/ French/ German | Ohrensausen |
|  | Ohrgeräusch(e) |
|  | Tinnitus |
| English/ Indonesian | Tinnitus |
| English/ Kinyarwanda/ French | Gusamira |
|  | Injereri |
|  | Urusaku |
| English/ Malay/ Tamil | ADA DENGAR BUNYI DESING |
| English/ Oriya/ Hindi | Beep |
| English/Greek | Buzzing |
|  | Hissing |
|  | Tinnitus |
| English/Hindi/ Malayalam | Hissing |
|  | Pulse like |
|  | Ringing |
| English/Spanish | Acufeno |
|  | Imagination |
|  | Pito |
|  | Tinitus |
|  | Tinnitus |
| Farsi/Persian | Soot |
|  | Vez vez |
| Flemish | fantoomgeluid |
|  | oorsuizen |
|  | Tinnitus |
| French | Acouphène |
|  | Hallucination auditive |
|  | Voix intérieure |
| French/ Arabic/ English | Acouphène |
|  | Tinnitus |
| French/ English | Acouphènes |
| French/ Italian | fischio continuo |
|  | ronzio continuo |
| German | Belastend |
|  | Nervig |
|  | Ohrgeräusch |
|  | Piepen |
|  | Tinnitus |
|  | Ton |
| German/ Dutch/ English | Ohrensausen |
|  | piepen im Ohr |
|  | Tinnitus (haben) |
| German/ English | Auditory hallucination |
|  | Ohrenrauschen |
|  | Piepen |
|  | Rauschen |
|  | Tinnitus |
| Greek | /emvo'es/ |
|  | Vouido (Greek word anglisized) |
| Gujarati/ English | Noise |
|  | Tinnitus |
| Hindi | Kaan baj rha hai |
|  | Kaan ke andar ki awaaz |
|  | Seeti jaisi awaaz aana |
|  | सीटी की अवाज आना। |
| Hindi/ English | Kaan bajna |
|  | Kaan me awaz |
|  | Kaan mein khuli aa rahi hai |
|  | Kaane shabda hoye |
|  | Something's ringing in my ear |
|  | Tinnitus |
| Hungarian | fulcsenges |
|  | fulzugas, |
|  | Tinnitus |
| Italian/ English | Acufene |
|  | Tinnito |
| Italian/ English/Spanish | Avere un acufene |
|  | Sentire un fischio costante |
| Japanese | Miminari |
|  | 何か音が聞こえて気になる |
| Kannada | \|kivi ollage eno sound barthidhe\| |
|  | \|silent agi iro time alli, eno shabha kellusthiruthe\| |
|  | Agnyatha Shabdha |
|  | Gothildheru shabdha |
|  | Horagina shabda illade, kui athava gush antha shabda kelsuthe |
|  | Kivi guyguduvike |
|  | Thiladheru shabdha |
|  | Tinnitus |
| Kannada/ English | Beep |
|  | beretara shabda |
|  | kuku |
|  | Tinnitus |
| Lithuanian | Spengti ausyse/galvoje |
| Malay/ English | Tinnitus |
| Malayalam | /chevijil moollicha/ |
|  | /kadal/ |
|  | Bhayankara sabdam |
|  | Chevi choolam vilikkuka |
|  | Chevi moolal |
|  | Chevikkullil moolal shabdham |
|  | Cheviyil moolal |
|  | Cheviyil ninn endhelum shabdham undo |
|  | Cheviyil ninn koo sound |
|  | Erappu |
|  | Koi koi |
|  | Manimuzhakam |
|  | Mathi |
|  | Moolal |
|  | Mozhakkam |
|  | Nirth |
|  | Oru vere tharathil ille sabdam |
|  | Ringing in ear |
|  | Shabdham |
|  | Shridikkan patanilla |
|  | Sound |
|  | Theere sugam illa |
|  | Vand moolunna shabdham |
|  | മൂളൽ |
| Malayalam/ English | Beep sound |
|  | Muzhakam cheviyil |
|  | Tinnitus |
| Malayalam/ English/ Kannada | Cheviyil ninnulla moolakkam (in malayalam) |
|  | Tinnitus |
| Marathi | Kaanatun aawaz yete |
|  | Kaan vajane |
|  | Kan vajne |
| Marathi/ English/ Hindi | कानाच्या आतुन आवाज ऐकू येतो का? |
|  | बाहेर कुठला ही आवाज नसताना, शांत असताना कानात शिट्टी किव्हा असा दडे बसल्यासारखे आवाज येतात का? |
| Nepali/ English | Kaan karauni samasya |
|  | Kaan ma awaj |
| Norwegian | Øresus |
|  | Piping |
|  | Tinnitus |
| Persian | vezooz |
|  | سوت کشیدن گوش |
|  | وز وز گوش |
| Persian/ Kurdish | Vezvez |
| Polish | dzwonienie |
|  | piszczenie |
|  | szumy uszne |
| Polish, English | Dzwonienie w uszach |
|  | Pisk w uszach |
|  | Szum w uszach |
| Portuguese | Alucinação auditiva |
|  | Apito |
|  | apito no ouvido |
|  | Ouvir um som ( beep,apito ou outro som) |
|  | Sem que ele corresponda a um som externo |
|  | Zumbido |
|  | Zumbido de abelha |
|  | zumbido no ouvido |
| Portuguese/ English | Apito |
|  | Chiado |
|  | Noise in the ear |
|  | Ouvindo coisas |
|  | Tinnitus |
|  | Zumbido |
| Romanian/ English | Ringing in the ears |
|  | Tinnitus |
| Russian | Звенит в ушах |
|  | звон в ушах |
|  | тиннитус |
|  | шум в ушах |
| Sinhalese | එක දිගට පවතින කන් අගුල් වැටීම |
|  | කන් අමාරුව |
|  | කන් කුරුකුරුව |
| Spanish | Acúfeno |
|  | Acufenos |
|  | chillido |
|  | Pitido |
|  | Tinnitus |
|  | Zumbido |
|  | Zumbidos |
| Spanish/ French | iiiii |
|  | Piiii |
|  | Tu tu tu |
| Tamil | Kaadhiraichal |
|  | Kaadhu iraichal |
|  | Kādhiraichal |
|  | Satham |
| Thai | ได้ยินเสียงดังในหู |
|  | เสียงดังในหูคล้ายจั๊กจั่นร้อง |
|  | เสียงดังรบกวนมากจนไม่มีสมาธิในการทำงาน |
| Turkish | Çınlama |
|  | Çıtırtı |
|  | Cızırtı |
|  | Gaipten sesler duymak |
|  | Kulak çınlaması |
|  | Tinitus |
| Turkish, English | Noise |
|  | Sounf |
| Urdu | Beenda |
|  | In my mother tongue I haven't come accros |
|  | Shoor |
| Urdu/ Punjabi | Minor rhythmic explosions with ringing |
|  | My heat is in my ears |
|  | Whooshing sound |
| Vietnamese/ English | Buzzing |
|  | Tinnitus |
